# The size of the packaging of ultra-processed cookies prevents them from having octagons despite their high sugar and saturated fat content

**DOI:** 10.17843/rpmesp.2023.403.13119

**Published:** 2023-09-28

**Authors:** Mayra Meza-Hernández, Kiomi Yabiku-Soto, Lorena Saavedra-Garcia, Francisco Diez-Canseco

**Affiliations:** 1 CRONICAS Center of Excellence in Chronic Diseases, Universidad Peruana Cayetano Heredia, Lima, Peru. Universidad Peruana Cayetano Heredia CRONICAS Center of Excellence in Chronic Diseases Universidad Peruana Cayetano Heredia Lima Peru

To the Editor. The regulation of the “Law for the Promotion of Healthy Eating for Children and Adolescents” (Law No. 30021) indicates that processed and ultra-processed foods and beverages that exceed the parameters for sugar content, saturated fat, sodium, and contain trans fats, must carry a frontal advertising warning (octagon) ^1^^,^^2^. The purpose of the octagons is to inform the population about the high content of these nutrients, so that they can make healthier choices when purchasing food.

Some processed and ultra-processed foods and beverages have been excluded from the aforementioned regulation and do not carry octagons because their packaging has a frontal area of less than 50 cm^2 (^^3^. These products are easily accessible to children and adolescents. In the first place, because, being small, they tend to have a more accessible price. In addition, they can be found frequently in school kiosks, street stalls, small stores and minimarkets, which are part of the school environment ^4^. Sweet cookies are among these products, so we sought to identify the proportion of cookies that exceed the parameters and do not have octagons due to the size of their individual packaging.

In May 2023, two nutritionists, members of the research team with experience in studies on ultra-processed foods, collected a sample of 75 ultra-processed cookies selected by convenience, in two minimarkets and a supermarket in Metropolitan Lima. They evaluated whether the content of sugar, saturated fat, sodium and the presence of trans fats exceeded the parameters established by Law No. 30021 based on the nutritional information declared on the label of their individual packages, in addition, the area of the front face of the individual packages was measured based on the guide for the placement of octagons published by the National Institute of Quality (INACAL) ^5^.

Ninety-two percent (69/75) of the ultra-processed cookies exceeded the amount of at least one of the four nutrients included in the established parameter. In addition, 53.6% (37/69) of the cookies that exceeded these parameters had the corresponding octagon. All cookies that exceeded at least one parameter and did not have the corresponding octagon on the individual packaging (n=32) exceeded the saturated fat parameter, and almost all exceeded the sugar parameter (96.9%). We found that 78.1% (25/32) did not have the warning label because the frontal area of their packaging was less than 50 cm^2^, but the remaining 21.9% (7/32) did have a frontal area greater than the established limit, thus failing to comply with the current regulation, as shown in Table 1. It is worth noting that 16.2% (6/37) of the cookies that had the corresponding octagons had a frontal area smaller than 50 cm^2^, which indicates that, despite not having the obligation, they had octagons in their individual packaging.

**Table 1:** Proportion of cookies without octagons in their individual packaging that exceed 50 cm^2^ of frontal area and each parameter regulated by Law 30021 (n=32).

Characteristic	Cookies without octagons in individual packaging
	n (%)
Front area of individual packaging	
Larger than 50 cm^2^	7 (21.9)
Less than 50 cm^2^	25 (78.1)
Number of cookies exceeding the parameters	
High sugar content	31 (96.9)
High saturated fats content	32 (100.0)
High sodium content	5 (15.6)
Contains trans fats	0 (0.0)

Our results show that many of the ultra-processed cookies offered in Metropolitan Lima do not have octagons on their individual packaging despite having high sugar and saturated fat contents because the frontal area of their packaging is less than 50 cm^2^. In this context, children and adolescents, who are the target population of Law 30021 ^1^, are exposed to ultra-processed foods with high sugar and saturated fat contents that are free of octagons due to the size of their packaging. This situation may cause misperceptions about the nutritional quality of these foods, showing them to be healthier than they actually are, in the eyes of children.

As in Peru, several Latin American countries have implemented frontal warnings in the form of octagons. Regarding small packages, Chile regulates that beverages and foods with a frontal area greater than 30 cm^2^ must have octagons ^6^. This allows the Chilean market to regulate a larger quantity of packaged products than the Peruvian market. On the other hand, Mexico and Argentina included the presence of octagons in small packages, placing a single octagon with a number, which indicates the total amount of octagons in this food ^7^^,^^8^. These experiences show that it is feasible to place octagons on small packages to inform the population of their contents.

In 2017, the draft Manual of Law No. 30021 established 20 cm^2^ as the size limit for the placement of octagons; however, this limit was increased to 50 cm^2^ the following year when the Manual was officially published ^9^. In this regard, the authorities of the Ministry of Health stated that this limit would be adjusted as the implementation of the Law progressed ^10^, however, as of June 2023 there has been no adjustment. Eliminating this limit on the size of the front packaging of processed and ultra-processed foods and beverages would allow all presentations of all products sold in the Peruvian market to be regulated. Thus, the use of octagons will allow the population, especially children and adolescents, to identify those beverages and foods free of octagons that do not exceed the parameters and, in this way, they can make healthier decisions when purchasing food ^11^. In addition, the presence of these warnings on all products would facilitate the monitoring of the proper use of octagons in all beverages and processed and ultra-processed foods in the Peruvian market.

One limitation of this study was that the sample is not representative of all ultra-processed cookies sold in the Peruvian market. In addition, the samples were collected in supermarkets and minimarkets, and not in other establishments that sell packaged foods.

In conclusion, our results show that almost all of the cookies sold in small packages have high sugar and saturated fat contents, but that many do not have the corresponding octagons on their individual packages because the frontal area of their packaging is less than 50 cm^2^, and others because they do not comply with the regulation. This reveals that the restriction of the use of octagons due to the size of the front package allows 78.1% of ultra-processed cookies that are high in sugar and saturated fats not to have octagons, which contravenes the right to information and the promotion of healthy habits, mainly in children and adolescents who are closer to small-sized products. It is important to carry out studies that expand the information presented in different categories of beverages and packaged foods.
