# Supplementary material for: The size of the packaging of ultra-processed cookies prevents them from having octagons despite their high sugar and saturated fat content
Source: Rev Peru Med Exp Salud Publica. 2023 Sep 28;40(3):369–71. doi: 10.17843/rpmesp.2023.403.13119 (PMC10953658; doi:10.17843/rpmesp.2023.403.13119)
Supplement: Translation

# Translation: El tamaño del empaque de las galletas ultraprocesadas evita que lleven octógonos a pesar de tener alto contenido de azúcar y grasas saturadas

**Authors:** Mayra Meza-Hernández, Kiomi Yabiku-Soto, Lorena Saavedra-Garcia, Francisco Diez-Canseco

**Affiliations:** 1 CRONICAS Centro de Excelencia en Enfermedades Crónicas, Universidad Peruana Cayetano Heredia, Lima, Perú.

Sr. Editor. La reglamentación de la «Ley de promoción de la alimentación saludable para niños, niñas y adolescentes» (Ley N.º 30021) indica que las bebidas y alimentos procesados y ultraprocesados que sobrepasan los parámetros para el contenido de azúcar, grasas saturadas, sodio, y contienen grasas trans, deben llevar una advertencia publicitaria frontal (octógono) ^1^^,^^2^. Los octógonos tienen la finalidad de informar a la población sobre el alto contenido de dichos nutrientes, para que puedan tomar decisiones más saludables al momento de adquirir sus alimentos.

Algunas bebidas y alimentos procesados y ultraprocesados han sido excluidos de la regulación antes mencionada y no llevan octógonos debido a que su empaque tiene un área frontal menor a 50 cm^2 (^^3^. Estos productos son de fácil acceso para niños, niñas y adolescentes. En primer lugar, porque al ser porciones pequeñas suelen tener un precio más accesible. En segundo lugar, se pueden encontrar, con más frecuencia, en quioscos escolares, puestos ambulantes, bodegas y *minimarkets*, que son parte del entorno escolar ^4^. Dentro de estos productos se encuentran las galletas dulces, por ello se buscó identificar la proporción galletas que superan los parámetros y no llevan octógonos debido al tamaño de su empaque individual.

En mayo del 2023, dos nutricionistas, miembros del equipo de investigación con experiencia en estudios sobre alimentos ultraprocesados, recolectaron una muestra de 75 galletas ultraprocesadas seleccionadas a conveniencia, en dos *minimarkets* y un supermercado de Lima Metropolitana. Se evaluó si el contenido de azúcar, grasas saturadas, sodio y la presencia de grasas trans superaban los parámetros establecidos por la Ley N.º 30021 basado de la información nutricional declarada en la etiqueta de sus empaques individuales, y se midió el área de la cara frontal de los empaques individuales en base a la guía para la colocación de octógonos publicada por Instituto Nacional de Calidad (INACAL) ^5^.

El 92% (69/75) de las galletas ultraprocesadas incluidas en el análisis, superaron la cantidad de, al menos, uno de los cuatro nutrientes evaluados respecto al parámetro establecido. Además, el 53,6% (37/69) de las galletas que superaban estos parámetros llevaban el octógono correspondiente. Del total de galletas que superaban al menos un parámetro y no llevaba el octógono correspondiente en el empaque individual (n=32), todas superaban el parámetro de grasas saturadas y casi todas el de azúcar (96,9%). El 78,1% (25/32) no llevaban la advertencia porque el área frontal de su empaque era menor a los 50 cm^2^, pero el 21,9% (7/32) restante si tenían un área frontal mayor al límite establecido, por lo que incumplían la regulación vigente, como se observa en la Tabla 1. Cabe resaltar que el 16,2% (6/37) de las galletas que llevaban los octógonos correspondientes tenía un área frontal menor de 50 cm^2^ lo cual indica que, a pesar de no tener la obligación, llevan octógonos en su empaque individual.

**Tabla 1:** Proporción de galletas sin octógonos en su empaque individual que superan los 50 cm^2^ de área frontal y cada parámetro regulado en la Ley 30021.

Característica	Sin octógonos en empaque individual (n=32)
	n (%)
Área frontal del empaque individual	
Mayor igual a 50 cm^2^	7 (21,9)
Menor a 50 cm^2^	25 (78,1)
Número de galletas que superan los parámetros	
Alto en azúcar	31 (96,9)
Alto en grasas saturadas	32 (100,0)
Alto en sodio	5 (15,6)
Contiene grasas trans	0 (0,0)

Nuestros resultados evidencian que muchas de las galletas ultraprocesadas que se ofertan en Lima Metropolitana no llevan octógonos en su empaque individual a pesar de tener altos contenidos de azúcar y grasas saturadas debido a que el área frontal de sus empaques es menor a 50 cm^2^. En este contexto, los niños, niñas y adolescentes, que son la población objetivo de la Ley 30021 ^1^, están expuestos a alimentos ultraprocesados con altos contenidos de azúcar y grasas saturadas que, se encuentran libres de octógonos por el tamaño de su empaque. Esta situación puede ocasionar percepciones equivocadas sobre la calidad nutricional de estos alimentos, mostrándolos como más saludables de lo que son, a los ojos de los y las menores.

Al igual que en el Perú, varios países latinoamericanos han implementado las advertencias frontales en forma de octógonos. En relación con los empaques pequeños, Chile regula que las bebidas y alimentos con un área frontal mayor a 30 cm^2^ deben llevar octógonos ^6^. Esto permite que en el mercado chileno se regule una mayor cantidad de productos envasados que en el peruano. Por otro lado, México y Argentina incluyeron la presencia de octógonos en envases pequeños, colocando un único octógono con un número, el cual indica la cantidad total de octógonos que lleva este alimento ^7^^,^^8^. Estas experiencias evidencian que es viable colocar octógonos en empaques pequeños para informar a la población de su contenido.

En 2017, en el proyecto de Manual de la Ley N.º 30021 se estableció 20 cm^2^ como límite de tamaño para la colocación de octógonos; sin embargo, al año siguiente en la publicación oficial del Manual este límite se incrementó a 50 cm^2 (^^9^^).^ Al respecto, las autoridades del Ministerio de Salud declararon que este límite se iba a ajustar conforme avance la implementación de la Ley ^10^, no obstante, hasta junio del 2023 no ha habido ajuste alguno. Eliminar este límite de tamaño del empaque frontal de las bebidas y alimentos procesados y ultraprocesados permitiría que todas las presentaciones de la totalidad de productos expendidos en el mercado peruano sean reguladas. Así, el uso de octógonos permitirá que la población, sobre todo los niños, niñas y adolescentes identifiquen las bebidas y alimentos libres de octógonos que realmente no superan los parámetros y que, de esta manera puedan tomar decisiones más saludables a la hora de adquirir alimentos ^11^. Además, la presencia de estas advertencias en la totalidad de productos facilitaría el monitoreo del uso adecuado de los octógonos en todas las bebidas y alimentos procesados y ultraprocesados del mercado peruano.

Una limitación de este estudio fue que la muestra no es representativa de la totalidad de galletas ultraprocesadas que se expenden en el mercado peruano. Además, la recolección fue realizada en supermercados y *minimarkets*, y no abarco otros establecimientos de venta de alimentos envasados.

En conclusión, nuestros resultados muestran que casi la totalidad de las galletas en presentaciones pequeñas tienen altos contenidos de azúcar y grasas saturadas pero que, muchas no llevan los octógonos correspondientes en sus empaques individuales porque el área frontal de su empaque es menor a 50 cm^2^, y otras por desobedecer la regulación, lo cual revela que la restricción del uso de octógonos por el tamaño del empaque frontal permite que el 78,1% de galletas ultraprocesadas que tienen alto contenido de azúcar y grasas saturadas no presenten octógonos, lo que contraviene el derecho a la información y la promoción de hábitos saludables, principalmente en los niños, niñas y adolescentes quienes tienen mayor cercanía a los productos de tamaño pequeño. Resulta relevante realizar estudios que amplíen la información presentada en diferentes categorías de bebidas y alimentos envasados.
